# Fusarivirus accessory helicases present an evolutionary link for viruses infecting plants and fungi

**DOI:** 10.1016/j.virs.2022.03.010

**Published:** 2022-03-18

**Authors:** Assane Hamidou Abdoulaye, Jichun Jia, Aqleem Abbas, Du Hai, Jiasen Cheng, Yanping Fu, Yang Lin, Daohong Jiang, Jiatao Xie

**Affiliations:** a State Key Laboratory of Agricultural Microbiology, Huazhong Agricultural University, Wuhan, 430070, China; b Hubei Key Laboratory of Plant Pathology, College of Plant Science and Technology, Huazhong Agricultural University, Wuhan, 430070, China; c Hubei Hongshan Laboratory, Wuhan, 430070, China

**Keywords:** Fusarivirus, Potyvirus, Hypovirus, Helicase, Horizontal gene transfer (HGT)

## Abstract

A significant number of mycoviruses have been identified that are related to plant viruses, but their evolutionary relationships are largely unexplored. A fusarivirus, Rhizoctonia solani fusarivirus 4 (RsFV4), was identified in phytopathogenic fungus *Rhizoctonia solani* (*R. solani*) strain XY74 co-infected by an alphaendornavirus. RsFV4 had a genome of 10,833 ​nt (excluding the poly-A tail), and consisted of four non-overlapping open reading frames (ORFs). ORF1 encodes an 825 aa protein containing a conserved helicase domain (Hel1). ORF3 encodes 1550 aa protein with two conserved domains, namely an RNA-dependent RNA polymerase (RdRp) and another helicase (Hel2). The ORF2 and ORF4 likely encode two hypothetical proteins (520 and 542 aa) with unknown functions. The phylogenetic analysis based on Hel2 and RdRp suggest that RsFV4 was positioned within the fusarivirus group, but formed an independent branch with three previously reported fusariviruses of *R. solani*. Notably, the Hel1 and its relatives were phylogenetically closer to helicases of potyviruses and hypoviruses than fusariviruses, suggesting fusarivirus Hel1 formed an evolutionary link between these three virus groups. This finding provides evidence of the occurrence of a horizontal gene transfer or recombination event between mycoviruses and plant viruses or between mycoviruses. Our findings are likely to enhance the understanding of virus evolution and diversity.

## Highlights

• *Rhizoctonia solani* strain XY74 hosts two mycoviruses, fusarivirus (RsFV4) and endornavirus (RsAEV1).
• RsFV4 consists of four ORFs and is evolutionarily associated to fusariviruses.
• Two ORFs of RsFV4 encode two helicases belonging to superfamly II.
• The accessory helicase of RsFV4 and its relatives are phylogenetically related to mycoviruses and plant viruses.

## 1 Introduction

Fungi and plants have formed dynamic reciprocal symbiotic relationships that are vital to the wellbeing of both partners; additionally, antagonistic interactions that play crucial roles in plant pathology have also been reported (Rodriguez and Redman, 2008; Roossinck, 2019). A range of potential gene transmissions has occurred between fungi and plants since wild plants are almost often colonized by endophytic, parasitic, and mycorrhizal fungi (Rodriguez et al., 2009; Roossinck, 2019). Fungi and plants are commonly infected by viruses that are at times phylogenetically related to each other (Liu et al., 2010; Donaire et al., 2016). Key examples include the fungus-infecting hypoviruses and fusariviruses that are evolutionarily related to plant potyviruses (Koonin et al., 1991). *Potyviridae* is the largest family of plant RNA viruses and contains positive-sense (+) single-stranded (ss) RNA genomes with flexuous filamentous particles (Wylie et al., 2017). A reasonable explanation for the frequent communication between plant and fungal viruses is their close relationship, and (+)ssRNA mycoviruses related to potyviruses may be inclined to lose capsid gene due to their intracellular lifestyle.

*Rhizoctonia solani* (*R. solani*; teleomorph: *Thanatephorus cucumeris*) is a basidiomycete phytopathogenic fungus responsible for the development of several crop diseases, which presents a wide host range and a remarkable geographical distribution (Xia et al., 2017). This phytopathogenic fungus causes diverse symptoms of infection depending on the host, including damping-off of seedlings, stem canker, and root or stem rot. Mycoviruses (or fungal viruses) are prevalent in all major fungi groups (Ghabrial et al., 2015; Son et al., 2015). Fusariviridae is a proposed family that comprises mycoviruses with (+)ssRNA genomes ranging from 6 to 7.7 kb (Zhang et al., 2014), and demonstrates complex genomic organizations. Certain fusariviruses contain two large open reading frames (ORFs) (Zhang et al., 2014; Liu et al., 2020), while others contain an additional one or two small ORFs (Kwon et al., 2007; Zhang et al., 2014; Liu et al., 2015; Hrabáková et al., 2017). The largest ORF codes for polymerase, including RdRp and helicase domains, whereas the second-largest and the small ORFs code for proteins of unknown function (Hrabáková et al., 2017). All known fusariviruses have been reportedly isolated from the ascomycetous fungi of classes Dothideomycetes, Leotiomycetes, and Sordariomycetes (Hrabáková et al., 2017). However, a metatranscriptomic analysis reported the detection of novel mycoviruses related to fusariviruses from the basidiomycete *Rhizoctonia solani* AG2-2 (Picarelli et al., 2019). Mycoviruses in *R. solani* were first described by Castanho (Castanho et al., 1978). To the best of our knowledge, few mycoviruses have been described so far infecting *R. solani* AG-1IA. So far, only two unclassified dsRNA mycoviruses (Zheng et al., 2013; Zhong et al., 2015), five parititiviruses (Zhang et al., 2018; Zheng et al., 2014; Lyu et al., 2018; Liu et al., 2018), an endornavirus (Zheng et al., 2019), and a hypovirus (Abdoulaye et al., 2021) were reported and characterized.

Helicases are nucleic acid-dependent ATPases capable of unwinding duplex DNA or RNA substrates *in vitro*, a critical step in genome replication, expression, recombination, and repair (Singleton et al., 2007). Nonetheless, they may perform additional functions *in vivo*, such as RNA annealing, clamping, and dissociation of RNA-bound proteins or RNA-protein complexes from RNAs (Halls et al., 2007; Yang et al., 2005; Ballut et al., 2005; Jankowsky et al., 2001; Bowers et al., 2006; Theuser et al., 2016). Helicases have been classified into six superfamilies (SF1–SF6) based on conserved motifs and comparative structural and functional analysis, with SF2 being the largest (Singleton et al., 2007; Fairman-Williams et al., 2010; Jankowsky, 2011; Gorbalenya, 1993). Despite their similar core domains, most helicases have variable flanking C and N-terminal extensions that contain additional domains for additional functions (Zhang and Grosse, 2004; He et al., 2010; Cui et al., 2008). Ring-shaped helicases are found in viruses and prokaryotes and belong to SF3–SF6. In contrast, eukaryotic helicases do not form the ring structure and belong to SF1 and SF2 (Jankowsky, 2011). RNA helicases are identified in all three major realms of life, and many viruses encode one or more of these proteins (Jankowsky, 2011; Gorbalenya, 1993). For instance, alphaendornaviruses and tymo-like viruses encode two helicases (Tuomivirta et al., 2009; Koonin et al., 1993). Helicase functions vary according to host. For instance, helicase defects are associated with premature aging and cancer predisposition in humans (Chu and Hickson, 2009; Larsen and Hickson, 2013); or a significant decrease in biofilms on abiotic surfaces and host leaves in bacteria (Granato et al., 2016).

We here discovered and characterized two (+)ssRNA mycoviruses, Rhizoctonia solani fusarivirus 4 (RsFV4) and Rhizoctonia solani alphaendornavirus 1 (RsAEV1), in the *R. solani* strain XY74 via metatranscriptomic analysis. Two helicases (Hel1 and Hel2) were detected in the genome of RsFV4, and phylogenetic analysis revealed potential evidence of the occurrence of a horizontal gene transfer (HGT) event triggering the transfer of genes between mycoviruses and plant viruses or mycoviruses. The RsFV4 effect was finally investigated in *R. solani*.

## 2 Materials and methods

### 2.1 Fungal isolation and culture conditions

Strain XY74 was isolated in Xiangyang County, Hubei Province, People's Republic of China, from rice plant tissues that exhibited rice sheath blight symptoms. *R. solani* strain 190, an RsAEV1- and RsFV4-free strain, was used as a negative control. All characterization experiments were qualitatively conducted in a laboratory. Strains XY74 and 190 were cultured in a petri dish containing potato dextrose agarose (PDA) at an optimum temperature of 28 ​°C and a photoperiod of 16 ​h/8 ​h (day/night). The internal transcribed spacer region was amplified using PCR to identify strain XY74 belonging to *R. solani* AG-1IA.

### 2.2 Nucleic acid extraction and purification

Double-stranded RNA (dsRNA) extraction was performed with cellulose, following a modified method (Xie et al., 2006). The fungal mycelia were first cultured on a sterilized cellophane membrane, and then placed onto a PDA plate (9 ​cm in diameter). The fresh mycelia (2–3 days post-inoculation, at 28 ​°C) were harvested and ground in liquid nitrogen. Subsequently, the extracted dsRNA was dissolved in ion-free water (ddH_2_O) or diethyl pyrocarbonate (DEPC) water and was subjected to treatment with S1 nuclease and DNase I (Takara, Dalian, China). Nucleic acid purification was performed using a gel extraction kit (Takara, Dalian, China) and stored at −80 ​°C prior to further utilization.

Total RNA isolation was conducted using the TRIzol Reagent Extraction Kit (Takara, Dalian, China) with slight modifications. Briefly, the fungal mycelia were ground to a fine powder in liquid nitrogen using a sterilized mortar and pestle. Subsequently, 1 ​mL of the TRIzol reagent (Takara, Dalian, China) was added to the mycelia powder. The mixture was maintained on ice for a few minutes until stratification was observed. The supernatant (containing total RNA) was precipitated at −20 ​°C using isopropyl alcohol or ethanol. The nucleic acid pellet was collected and subjected to washing steps using 70% ethanol and dissolved in ddH_2_O or DEPC water. The purified RNA was used for conducting reverse transcription (RT) PCR to amplify cloned DNA samples and was subjected to whole-genome sequencing.

### 2.3 High-throughput sequencing

The Shanghai Biotechnology Corporation conducted high-throughput sequencing of the samples using a high-throughput sequencer (Hiseq 2000/2500, Illumina, San Diego, CA, USA). The sequencing library was constructed using the TruSeq™ RNA Sample Prep Kit (Illumina RS-122-2001). However, sequencing results in the obtainment of raw reads containing unqualified reads with the low overall quality, sequencing primers, and low-end quality. Therefore, the total reads were filtered with Trimmonatic (Version 0.36) using the following procedure: (i) readings with the lowest overall quality and read with a mass greater than 20 bases and less than 50% were removed; (ii) the bases at the 3′ end mass Q below 20 were removed, the base error rate was <0.01, where Q ​= ​−10 log^error_ratio^; (iii) the linker sequence present in the reads was removed; (iv) ambiguous N bases present in the reads due to insufficient sequencing intensity and unrecognizable bases were removed; (v) sequencing fragments (lengths <20) were removed; and (vi) all rRNA and mRNA sequences derived from the host were removed. *De novo* assembly of the qualified reads was conducted using a Scaffolding contig algorithm developed by CLC Genomics Workbench (version: 6.0.4) (Bräutigam et al., 2011; Garg et al., 2011; Su et al., 2011). Data on the first sequence splicing, termed as primary UniGenes, were obtained. Primary UniGenes were then subjected to splicing twice using the CAP3 EST software, to obtain first and second contigs, respectively. The contigs obtained were analyzed using online tools such as National Center for Biotechnology (NCBI) Blastx and Protein BLAST (https://blast.ncbi.nlm.nih.gov/Blast.cgi).

### 2.4 Virus complete genome amplification

The 5′ and 3′ end regions were amplified as previous methods described by Potgieter et al. (2009), with minor modifications. The extracted dsRNA was first subjected to gel purification following the AxyPrep™ Nucleic Acid Purification Kit's instructions (AXYGEN, Suzhou, China). The terminal sequences of the purified dsRNA were ligated with the PC3-T7 loop (5′-p-GGATCCCGGGAATTCGGTAATACGACTCACTATATTTTTATAGTGAGTCGTATTA-OH-3′) by mixing and establishing a system comprising BSA, Recombinant RNase Inhibitor, T4 RNA ligase 40 U/μL, 10 ​× ​T4 RNA buffer (Takara, Dalian, China), 50% PEG 6000 and supplemented with ddH_2_O. The mixture was incubated at 4 ​°C for 18 ​h. Following the denaturation of the purified ligated dsRNA with Dimethyl sulfoxide, the denaturated dsRNA was thereafter cloned via RT-PCR. The resultant cDNA was subsequently amplified with 5′-GTGGCAAAACACCCGAAGAC-3′ and 5′-CTCCGAGCGTAGTTTGGGTT-3′ for the 3′ and 5′ ends, respectively, as the forward primers, and a complementary sequence to PC3-T7 loop, PC2 (5′-p-CCGAATTCCCGGGATCC-3′) as the reverse primer. The amplicons obtained were subsequently subjected to gel purification (Takara, Dalian, China), cloned in a vector pMD18-T (Takara, Dalian, China), and sequenced. At least four-time repetitions were performed during the 3′ and 5′ end amplification.

### 2.5 Sequences, phylogenetic analysis, and secondary structure prediction

Nucleotide sequence assembly and protein translations were performed using DNAMAN X version 10.3.3.102 and Geneious 5.6.5 (Kearse et al., 2012; Wang, 2015). To determine and analyze the putative proteins, different tools were used, including ORFfinder tool (http://www.ncbi.nlm.nih.gov/projects/gorf), motif search (https://www.genome.jp/), and Transmembrane alpha-helices using the TMHMM (https://services.healthtech.dtu.dk/). Multiple alignments was conducted to determine the characteristic of the helicases conserved motifs with MAFFT (Version 7.427) using the E-INS-i model and trimmed with trimAl (Version 1.4) (Katoh and Standley, 2013; Capella-Gutiérrez et al., 2009). Phylogenetic tree analysis of helicases domain was efficiently constructed using IQ-TREE (Version 1.6.11) with 1000 bootstrap replications (Minh et al., 2020). Furthermore, multiple alignments were performed using the ClustalW algorithm to determine the RdRp conserved domain motifs. The phylogenetic tree of RdRp was constructed using the MEGAX program, by applying the maximum likelihood (ML) method with substitution model LG+G+I and 1000 bootstrap replications. The best-fit amino acid substitution models were identified using ModelFinder (Kalyaanamoorthy et al., 2017).

### 2.6 Protoplasts and hyphae tip isolation

A single protoplast was isolated to determine the effect of mycoviruses in strain XY74 using the procedure described by Feng et al. (2012) with minor modifications. The fresh mycelia were collected from a sterilized cellophane membrane placed onto 9-cm PDA plates, and then incubated in flasks supplemented with 100 ​mL of potato dextrose broth (PDB) and subjected to a speed shaking condition at 4×*g* at 28 ​°C for 12 ​h. The mycelia were ground with a sterilized mortar and pestle, and then suspended into a fresh PDB medium. The mycelia were harvested by filtering them through gauze layers, and then they were subjected to washing steps using osmoticum (0.7 ​mol/L MgSO_4_). The mycelia were resuspended in a filter-sterilized enzyme mixture, composed of 1% (w/v) cellulase (Sigma-Aldrich, St Louis, MO, USA), 1% (w/v) driselase (Sigma-Aldrich, St Louis, MO, USA) and 0.1% (w/v) (lysing Enzymes Sigma-Aldrich, St Louis, MO, USA), and were subjected to shaking conditions at 28 ​°C for 3 ​h to obtain a uniform distribution. The mixture was then filtered through a 120-μm pore nylon mesh. The protoplast suspension was subjected to speed centrifugation at 1200 ×*g* for 10 ​min, washed twice with osmoticum, and resuspended in an appropriate Sorbitol Tris Calcium volume (1 ​mol/L sorbitol, 50 ​mmol/L Tris-HCl pH ​= ​8, 50 ​mmol/L CaCl_2_). The resulting protoplasts were determined using a microscope and a hemocytometer, and measurements were acquired every half an hour until a considerable number of protoplasts were released. Lastly, the resulting protoplasts were spread onto the regeneration medium surface and cultivated at 28 ​°C to obtain the regeneration rate of the protoplasts.

Mycelia plugs were grown on PDA plates at 28 ​°C for one day, under conditions of a 16-h photoperiod. Single hyphae tips were cut with a sterilized needle and inoculated onto fresh PDA for additional cycles. At least seven repetitions were conducted during this final procedure to prevent the obtainment of inaccurate results. All regenerated isolates were subjected to mycelial colony morphology and dsRNA isolation analyses to detect the mycovirus genome.

### 2.7 Mycovirus transmission and virulence assays

Mycoviruses were horizontally transmitted via hyphal anastomosis. The strains XY74 (donor) and 190 (recipient) were first cultured on 9-cm PDA plates. The mycelial agar plugs obtained from selected strains were then cultured on PDA plates. Subsequently, the mycelial agar plugs were removed over a time interval ranging from 48 to 96 ​h, after both colonies exhibited expansion and established contact. Samples were acquired from the sides exhibiting growth of the recipient strain 190 and from locations distant from the contact line formed between the two colonies. All fresh isolates were re-cultured on PDA plates. The horizontal transmission assay was repeated at least three times to avoid the obtainment of inaccurate results. Furthermore, the new virus-infected isolates were subjected to dsRNA isolation and mycovirus detection through RT-PCR amplification.

We conducted a pathogenicity experiment on the detached rice leaves to assess the virulence of *R. solani* XY74 strain. Fungal mycelial plugs were inoculated on the detached rice leaves and were incubated in a growth chamber at 28 ​°C. The procedure was repeated at least three times.

### 2.8 Mycovirus detection by northern blotting

Northern blotting results were appropriately assessed to confirm the presence of RsAEV1 and RsFV4 in strain XY74. Briefly, dsRNA molecules were size-fractionated on 1% (w/v) TAE agarose (40 ​mmol/L Tris/acetic acid, 1 ​mmol/L EDTA, pH 7.6), and gel electrophoresis was performed for 2–4 ​h at 4 ​°C. Subsequently, the gel was soaked in 0.1 ​mol/L NaOH for 20 ​min and then in 0.1 ​mol/L Tris/HCl at pH 8.0 for 20 ​min. Thereafter, dsRNA molecules were transferred onto a transfer membrane (Amersham Hybond-N^+^ nylon, GE Healthcare, Shanghai, China) in a 10 ​× ​SSC buffer via capillary action along with the RNA that was covalently bound (Jiang and Ghabrial, 2004). Thereafter, the transfer membrane was analyzed by conducting hybridization with a specific probe prepared using PCR (GE Healthcare, RPN3682). The designed specific primers RsFV4-F1 5′- TCTTGTTTCCAGCGAGACCC -3′ and RsFV4-R1 5′- GTTGGTGTGACGCTACCTGA -3′ were utilized to prepare probes for RsFV4-F1; and RsAEV1-F1 5′-TACATGCCCGCATTGGAGAG-3′ and RsAEV1-R1 5′-GCGTTCTGTATGCCACGTTC-3′ for RsAEV1. RsFV4 RNA hybridization, using filter paper membrane transfer, was confirmed by autoradiography using the GEL Doc™ EZ Imager Fluorescent Image Analyzer (BIO-RAD, Shanghai, China).

## 3 Results

### 3.1 Two (+)ssRNA mycoviruses co-infect a single strain of *R. solani*

**Fig. 1:**
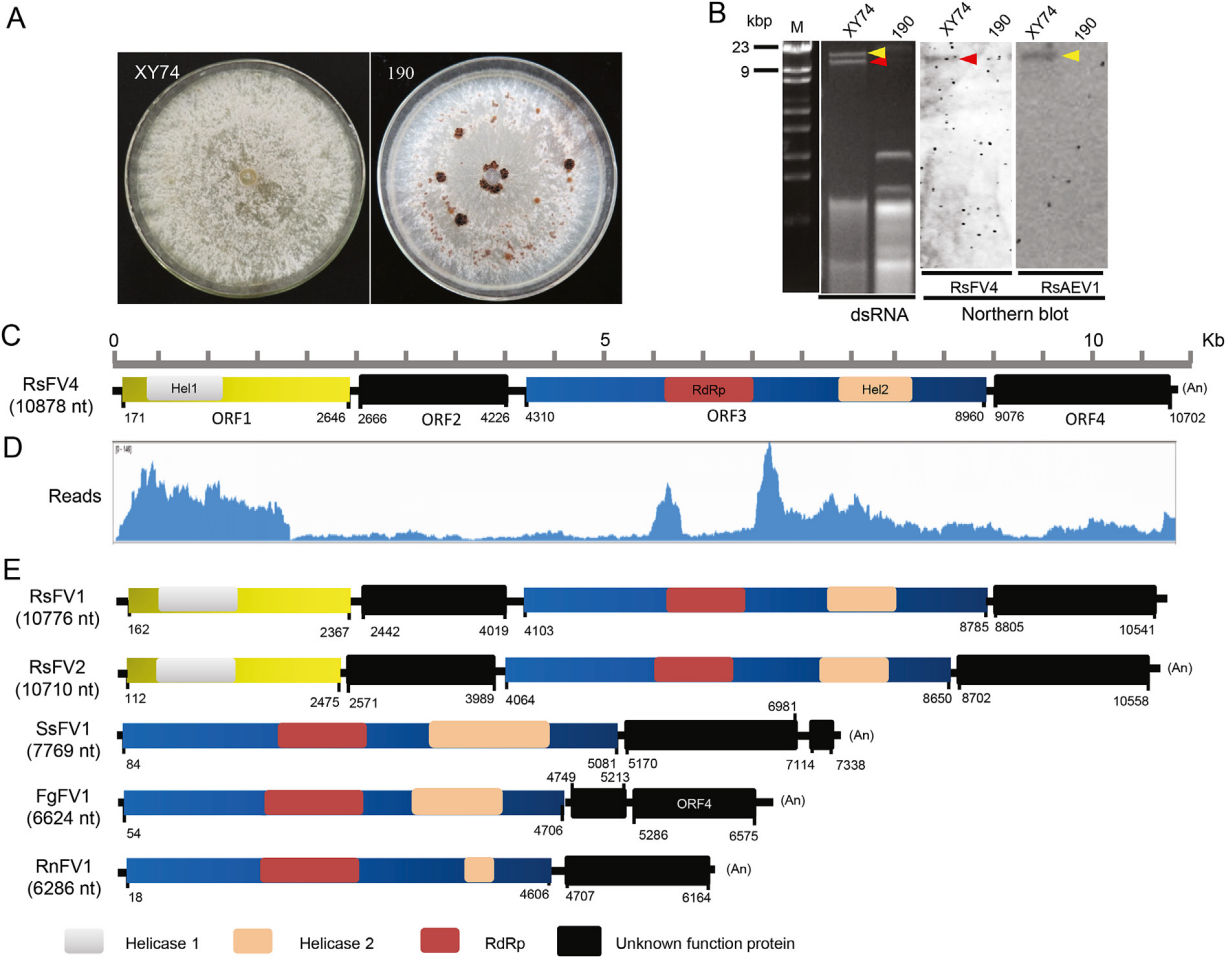
The molecular characterizations of Rhizoctonia solani fusarivirus 4 (RsFV4). **A** Colony morphology of strains XY74 and 190 on PDA medium. Pictures were taken after seven days of growth at 28 ​°C. **B** dsRNAs extraction and northern hybridization confirmation. The strain 190 is the RsFV4-free strain. Lane M: DNA molecular weight marker. The second lane contains XY74 dsRNA segments highlighted with yellow and red arrows that correspond to RsAEV1 and RsFV4, respectively. **C** Schematic genome organization of RsFV4. RsFV4 contains four non-overlapping ORFs (ORF1, ORF2, ORF3, and ORF4). The smaller ORF2 (520 aa) and ORF4 (542 aa) encode a putative protein of unknown function. The larger ORF3 (1550 aa) encodes a polyprotein of RNA dependent RNA polymerase (RdRp; pfam01699, E-value ​= ​3e-17) and HrpA-like RNA helicase (HrpA; cl34328, E-value ​= ​3e-07), while ORF1 encodes HrpA (cl34328, E-value ​= ​2.43e-08). **D** The distribution profile of transcriptomic reads and its maping against genome sequences of RsFV4. **E** Comparative genome organization of three *R. solani* fusariviruses and other representative fusariviruses. Schematic representation of the genome organizations and locations of each putative open reading frame. The conserved domains of helicase and RdRp were shown with different color frame. The members within the proposed family Fusariviridae have a complex and diverse genomic organization with two to four ORFs of different length. *R. solani* fusariviruses have two helicase domains, while other fusariviruses harbor a single helicase domain. RsFV4, Rhizoctonia solani fusarivirus 4; RsFV1, Rhizoctonia solani fusarivirus 1; RsFV2, Rhizoctonia solani fusarivirus 2; RnFV1, Rosellinia necatrix fusarivirus 1; FgFV1, Fusarium graminearum fusarivirus 1; SsFV1, Sclerotinia sclerotiorum fusarivirus 1.

The investigation of *R. solani* strain XY74 mycoviruses was undertaken owing to the exhibition of abnormal phenotypic traits (for instance, defective in sclerotium formation) (Fig. 1A). The dsRNAs were extracted and screened from the fungal mycelia in the nucleic acid extracts, to investigate whether mycoviruses infected strain XY74. The results revealed that the strain XY74 harbored multiple dsRNA segments of more than 9 kb in size (Fig. 1B). To elucidate the composition of mycoviruses in strain XY74, a targeted next-generation sequencing approach was used. Data analysis and sequence assembly revealed the existence of Contig_1 (3,779 reads) and Contig_13 (1,977 reads) of 16,636 ​nt and 10,815 ​nt in length, respectively. A homology search of the assembled sequences against NCBI protein databases showed similarity to alphaendornaviruses and fusariviruses, respectively. Contig_1 and Contig_13 were subsequently completely sequenced and named Rhizoctonia solani alphaendornavirus 1 (RsAEV1) and Rhizoctonia solani fusarivirus 4 (RsFV4), respectively.

A northern blot analysis was conducted using two mycovirus-specific probes to investigate RsAEV1 and RsFV4. As expected, RsAEV1 and RsFV4 were demonstrated to infect the strain XY74, whereas they were absent in the control strain 190 (Fig. 1B). Therefore, these results confirmed that strain XY74 was co-infected by RsAEV1 and RsFV4.

### 3.2 RsFV4 encodes two helicase genes locating in two ORFs

The designed primers (Supplementary Table S1) were utilized to perform sequencing of the entire genome and to confirm the accuracy of the RsFV4 sequence obtained via metatranscriptomic analysis. Subsequently, terminal sequences of RsFV4 were determined using the Rapid Amplification of cDNA Ends (RACE) technique. The RsFV4 complete genome consists of 10,833 ​nt, excluding the poly-A tail (Fig. 1C), and its genome has been deposited in GenBank under the accession number MW149059. The read mapping against RsFV4 genome sequences was conducted, and results suggested that reads could be well-matched to RsFV4 genome, but reads abundance from ORF1 and ORF3 were higher obviously than that from the other two ORFs in RsFV4 (Fig. 1D).

RsFV4 contained a rich A ​+ ​U content of 57.8%. RsFV4 protein translation revealed four ORFs (ORF1 to ORF4) localized on the positive strand. Sequence analysis of ORF1 to ORF4 showed an initiation codon (AUG) at positions 171, 2,666, 4,310, and 9076 ​nt, respectively. The smaller ORF2 and ORF4 showed a stop codon (UAG) at positions 4228 and 10,704 ​nt, respectively, while ORF1 and ORF3 showed a stop codon (UAA) at position 2648 and 8962 ​nt, respectively.

Motif search revealed that the smaller ORF2 (504 aa, 59.5 ​kDa) and ORF4 (542 aa, 60.6 ​kDa) encoded two hypothetical proteins with unknown functions. The larger polyprotein (1550 aa, 176.7 ​kDa) encoded by ORF3 included two conserved domains of RdRp (pfam01699, E-value ​= ​7e-21) and HrpA-like RNA helicase (Hel2, cl34328, E-value ​= ​2.43e-08). The putative ORF1-encoded protein contained another conserved HrpA-like RNA helicase domain (Hel1, cl34328, E-value ​= ​3e-07).

Blastx analysis of RsFV4 complete genome showed similarity to fusariviruses, including Rhizoctonia solani fusarivirus 1 (RsFV1; QDW92695, identity 42%, E-value ​= ​0.0), Rosellinia necatrix fusarivirus 2 (RnFV2; BBB86790, identity 24%, E-value ​= ​0.0), and Sclerotinia sclerotiorum fusarivirus 1 (SsFV1; YP_009143301, identity 34%, E-value ​= ​3e-179). Furthermore, Blastx analysis of the RsFV4 genome showed similarity with members within *Potyviridae*, and *Hypoviridae*. The putative proteins encoded by RsFV4 showed lower identities to other known viruses, except RsFV1 and RsFV2 (Table 1). Moreover, comparative genome organization of fusariviruses suggested that RsFV4 is similar to fusariviruses in *R. solanis*, but significant different from other known fusariviruses including SsFV1, RnFV1, and Fusarium graminearum fusarivirus 1 (FgFV1) in genome structure and size (Fig. 1E).

**Table 1:** The information of RsFV4 and its related viruses, and sequence identities (%) between RsFV4 and its related sequences based on the multiple alignments of the amino acid (aa) of different conserved domains.

Family	Virus name	Acronym	Genome length^a^	ORF^b^	Hel1 Ident. (%)^c^	ORF2-protein Ident. (%)^d^	RdRp Ident. (%)^e^	Hel2 Ident. (%)^e^	ORF4-protein Ident.(%)^d^	Accession Number
***Fusarivirdae***	Rhizoctonia solani fusarivirus 4	RsFV4	10,833	4	100	100	100	100	100	MW149059
	Rhizoctonia solani fusarivirus 1	RsFV1	10,776	4	53.75	26.00	78.09	65.06	23.00	MK558257
	Rhizoctonia solani fusarivirus 2	RsFV2	10,710	4	29.81	14.90	69.84	45.35	18.90	MK558256
	Alternaria brassicicola fusarivirus 1	AbFV1	6,656	3	12.55	/	38.93	27.54	/	NC_029056
	Aspergillus ellipticus fusarivirus 1	AeFV1	6,253	2	13.23	/	39.84	24.91	/	MK279500
	Fusarium graminearum mycovirus 1	FgMV1	6,624	4	13.93	/	39.84	37.37	/	NC_006937
	Fusarium poae fusarivirus 1	FpFV1	6,379	2	13.19	/	40.64	27.27	/	NC_030868
	Gaeumannomyces tritici fusarivirus 1	GtFV1	6,332	2	15.08	/	40.64	27.88	/	MK279501
	Morchella importuna fusarivirus 1	MiFV1	7,835	3	11.85	/	45.63	27.17	/	MK279502
	Neofusicoccum luteum fusarivirus 1	NlFV1	6,244	2	12.03	/	40.24	26.37	/	KY906214
	Neurospora discreta fusarivirus 1	NdFV1	6,648	2	10.99	/	41.04	26.71	/	MK279503
	Nigrospora oryzae fusarivirus 1	NoFV1	7,018	2	9.87	/	41.35	28.03	/	NC_031960
	Rosellinia necatrix fusarivirus 1	RnFV1	6,286	2	14.17	/	39.84	24.00	/	NC_024485
	Rutstroemia firma fusarivirus 1	FfFV1	6,641	2	14.36	/	40.24	23.27	/	MK279504
	Sclerotinia sclerotiorum fusarivirus 1	SsFV1	7,769	4	12.17	/	39.84	26.33	/	NC_027208
	Sclerotium rolfsii fusarivirus 1	SrFV1	7,301	2	13.75	/	39.84	25.72	/	MH766491
	Sodiomyces alkalinus fusarivirus 1	SaFV1	6,252	2	12.86	/	38.25	25.00	/	NC_040529
	Zymoseptoria tritici fusarivirus 1	ZgFV1	5,969	2	12.65	/	38.25	24.91	/	MK279506
***Hypoviridae***	Botrytis cinerea hypovirus 1	BcHV1	10,250	1	13.03	/	29.08	18.07	/	MH347277
	Criphonectria Hypovirus 3	CHV3	9,591	1	14.15	/	26.72	17.93	/	AF188514
	Criphonectria Hypovirus 4	CHV4	9,149	1	13.49	/	29.08	14.24	/	NC_006431
	Setosphaeria turcica hypovirus 1	StHV1	9,069	1	14.77	/	18.18	17.99	/	MK279474
	Trichoderma harzianum hypovirus 1	ThHV1	11,251	2	13.47	/	20.98	16.71	/	MN172262
	Valsa ceratosperma hypovirus 1	VcHV1	9,543	1	12.66	/	28.54	15.85	/	NC_017099
***Potyviridae***	Potato virus Y	PVY	9,704	1	16.08	/	14.12	12.61	/	NC_001616.1
	Plum pox virus	PlPV	9,741	2	13.82	/	14.02	13.09	/	NC_001445.1
	Potato virus V	PVV	9,848	2	16.52	/	13.61	15.59	/	NC_004010.1
	Tobacco mosqueado virus	TMV	9,896	1	17.99	/	14.50	10.73	/	NC_030118.1

“/” Undertermined; a, complete genome size (nt); b, the number of ORF in viruses; c, conserved helicase domain of protein encoded by ORF1 of RsFV4; d, Protein encoded by ORF2 or ORF4 of RsFV4; e, the RdRp and helicase domains in polyprotein encoded by ORF3 of RsFV4.

**Fig. 2:**
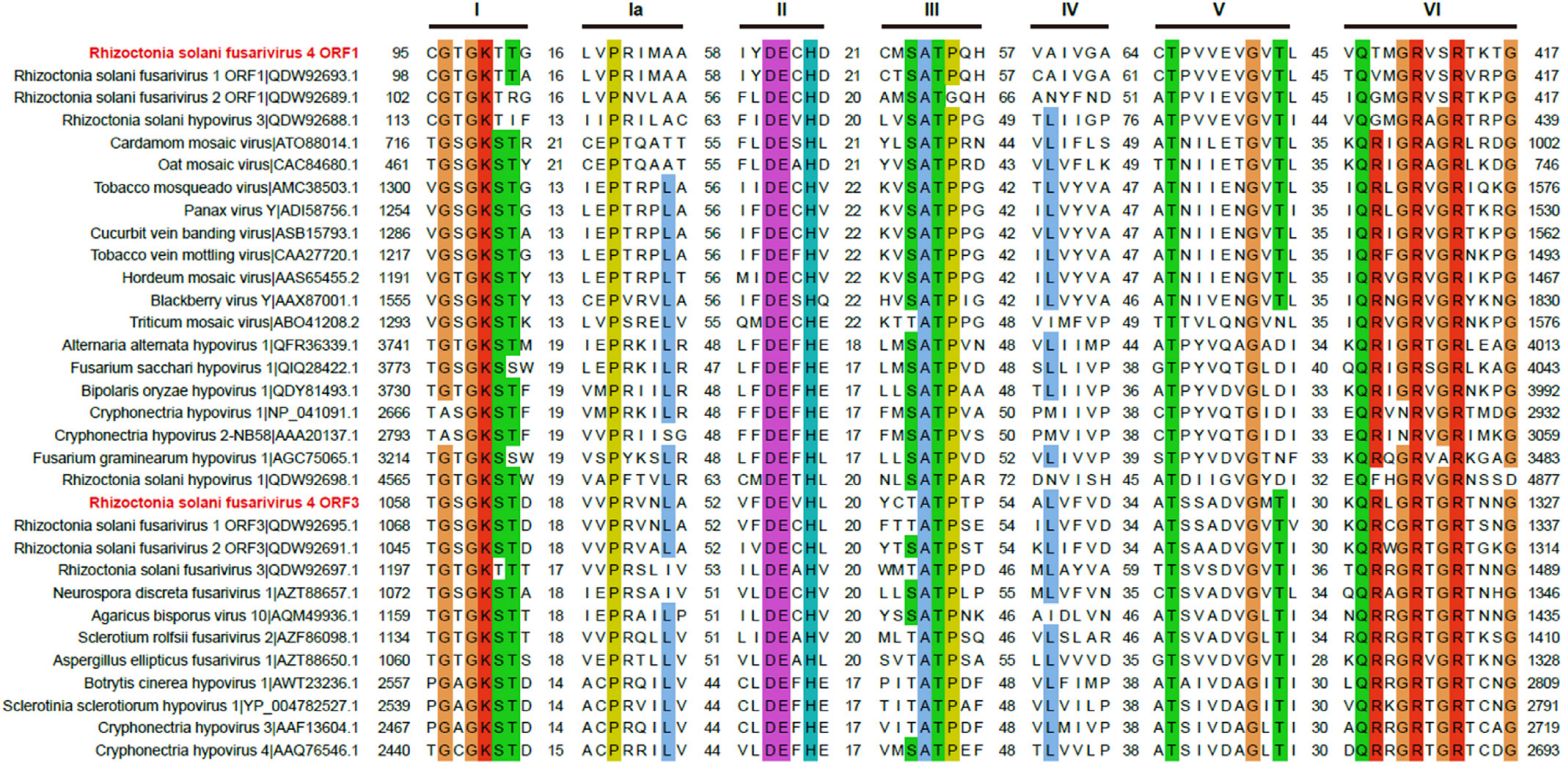
Multiple alignments of helicases in RsFV4 and its relatives. Alignment of two helicase domains of RsFV4 with their counterparts derived from viruses in the families *Hypoviridae*, *Potyviridae*, and the proposed family Fusariviridae. Seven motifs (I, Ia-VI) were detected in the sequence of the conserved helicase region. The default color scheme for ClustalW alignment in the Jalview program was used. Numbers within sequences correspond to the number of amino acid residues separating the motifs. Amino acid positions are indicated from the start and the stop regions of the ORF-encoded proteins.

Multiple sequence alignments of Hel1 and Hel2 showed the presence of both seven (I, Ia, and II-VI) domain-containing motifs demonstrated for the (+)ssRNA putative helicase superfamily (SF) II (Fig. 2). Motifs I and II contained NTP-binding motif, essential for virus reproduction (Koonin et al., 1993). Additionally, multiple sequence alignment of ORF3-encoded RdRp showed the presence of eight (I–VIII) domain-containing motifs demonstrated for the (+)ssRNA putative RdRp (Supplementary Fig. S1). The importance of the core RdRp motifs (IV, V, and VI) was demonstrated by site-directed mutagenesis of the Encephalomyocarditis virus polymerase (Sankar and Porter, 1992).

Two transmembrane (TM) domains were predicted at the C-terminal region of proteins encoded by ORF2 and ORF4 (Supplementary Fig. S2), whereas was found at the N-terminal region of protein encoded by ORF3 (Supplementary Fig. S2) that was similar to proteins encoded by ORF1 of FgFV1 and RnFV1. However, the coiled-coil domain was not detected in RsFV4, found in FgFV1 and RnFV1 (Kwon et al., 2007; Zhang et al., 2014). No TM domain was detected in proteins encoded by ORF1. Taken together, RsFV4 was proposed to be a (+)ssRNA mycovirus.

### 3.3 RsFV4 is phylogenetically related to fusariviruses, potyviruses, and hypoviruses

**Fig. 3:**
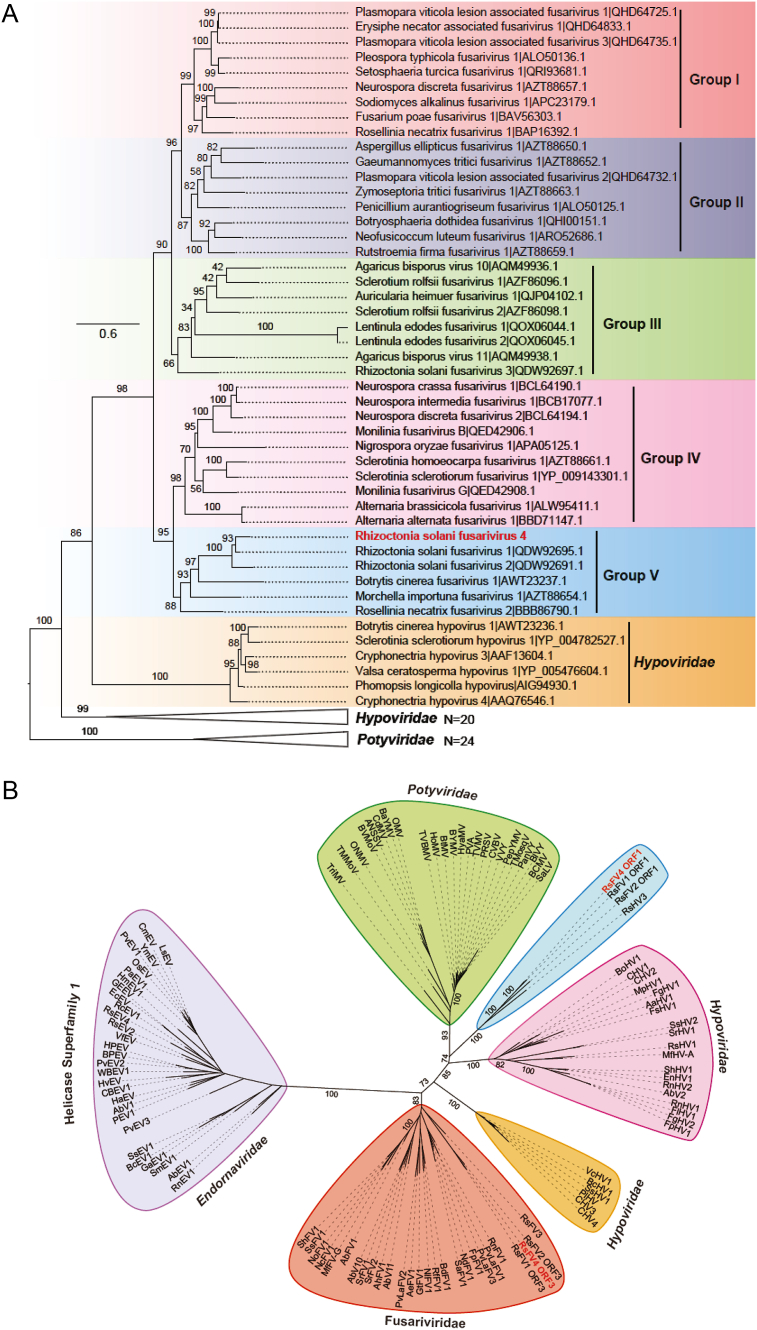
Phylogenetic analysis of the putative RsFV4 RdRp and helicases along with other related viruses. **A** Branch lengths are scaled to the expected underlying number of amino acid substitutions per site. The alignments were performed using the ClustalW algorithm, and the phylogenetic tree was constructed using the MEGAX program, by applying the maximum likelihood (ML) method with substitution model LG+G+I and 1000 bootstrap replications. The fusarivirus clade has been divided into five groups (I to V) and accession numbers follow virus names. Viruses within *Hypoviridae and Potyviridiae* are included as an outgroup. Bootstrap values (%) obtained with 1000 replicates are indicated on branches and branch lengths correspond to genetic distance; scale bar on the left corresponds to a genetic distance of 0.6. The Rhizoctonia solani fusarivirus 4 is highlighted in red color. **B** The phylogenetic tree was constructed using IQ-TREE with the best-fit model “Blosum62+F+R7.” Bootstrap values (%) obtained with 1000 replicates are indicated on branches and branch lengths correspond to genetic distance. The alignments were performed with MAFFT (Version 7.427) using the E-INS-i model and trimmed with trimAl. Selected viruses in families *Hypoviridae*, *Potyviridae*, and viruses that contain helicase belonging to superfamily II, were included in the phylogenetic tree. The RsFV4 is highlighted in red. Sequence information of all selected viruses has been provided in Supplementary Table S2.

An ML phylogenetic tree was constructed to investigate RsFV4 and the evolutionary relatedness of other known viruses. The conserved RdRp domain of RsFV4 shared high similarity to RsFV1 and RsFV2 with 78.1% and 69.8% identities (Table 1), respectively. Phylogenetic analysis based on multiple sequence alignment of the conserved RdRp regions derived from the referenced viruses helped categorize all known fusariviruses into five groups (I, II, III, IV, and V). RsFV4 clustered with five previously reported fusariviruses to form the group V (Fig. 3A), suggesting that RsFV4 should be considered a new taxon within the proposed family Fusariviridae.

*R. solani* fusariviruses (RsFV1, RsFV2, and RsFV4) were clustered in a well-supported branch based on helicase tree topology (Fig. 3B). The phylogenetic analysis of helicases showed that Hel1 and Hel2 were clustered into two distant clades, and both belong to the helicase supergroup II. Similar results of RdRp analysis, Hel2 of RsFV4 was positioned into the clade with the previously reported fusariviruses, but formed an independent phylogenetic group with three putative fusariviruses (RsFV1, RsFV2, and RsFV3) infecting *R. solani* (Fig. 3B). Unexpectedly, Hel1 phylogenetic analysis did not share similarities with other reported taxa in fusariviruses, with the exceptions of RsFV1 and RsFV2. The Hel1 of RsFV4 with the other two mycoviruses (RsFV1 and RsFV2) was phylogenetically related to members in families *Potyviridae* and *Hypoviridae* rather than fusariviruses group. These results revealed that fusariviruses in *R. solani* have a unique evolutionary process and provide evidence of the occurrence of HGT between mycoviruses (fusariviruses) and plant viruses (potyviruses) or mycoviruses (hypoviruses). A combination of the RdRp phylogenetic analysis supported the hypothesis that RsFV4 clustered with fusariviruses and showed marked similarity with viruses within families *Hypoviridae* and *Potyviridae* (Fig. 3A). In summary, these results suggest that RsFV4 is a novel (+)ssRNA mycovirus that is related to fusariviruses. Notably, an unclassified hypovirus, Rhizoctonia solani hypovirus 3 (RsHV3), clustered with RsFV1, RsFV2, and RsFV4 (Fig. 3B), suggesting that RsHV3 did not belong to family *Hypoviridae*; additionally, it exhibited a similar genome structure and evolutionary progress to RsFV4 and its relatives (Supplementary Fig. S2).

### 3.4 RsFV4 and RsAEV1 exert no noticeable impact on *R. solani*

**Fig. 4:**
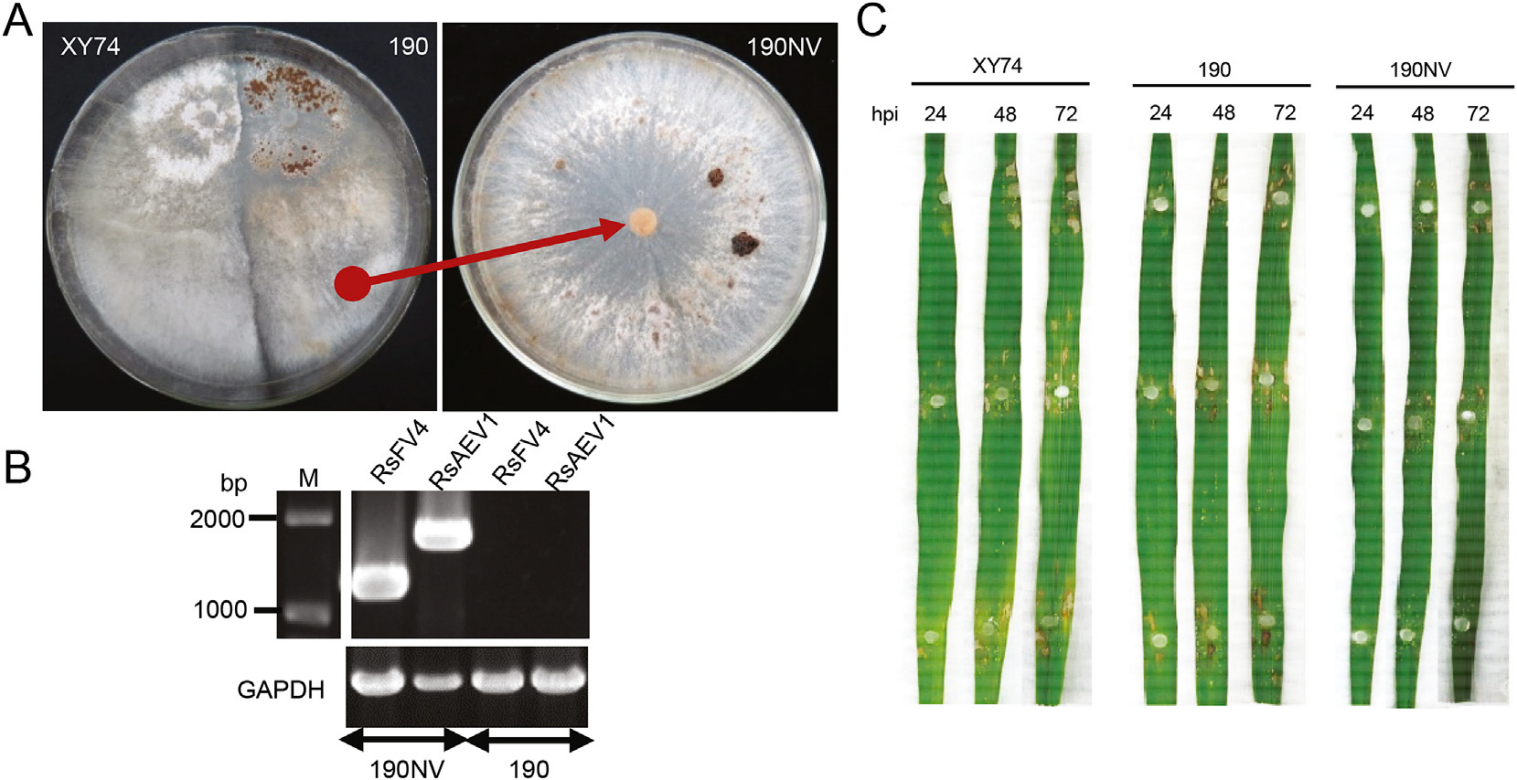
The impact of RsFV4 and RsAEV1 on *R. solani*. **A** Right plate: dual culture of *R. solani* strains XY74 and 190 on a PDA medium at 28 ​°C. Left plate: colony morphology of 190NV that is a new virus-infected isolates picked up from strain 190 side after dual-cultured (red arrow). Pictures were taken at 7 days post inoculations. **B** PCR detection of Rhizoctonia solani fusarivirus 4 (RsFV4), Rhizoctonia solani alphaendornavirus 1 (RsAEV1) using specific designed primers RsFV4-F1/R1 and RsAEV1-RsAEV1-F1/R1 (Supplementary Table S1). *GAPDH* (Glyceraldehyde-3-Phosphate Dehydrogenase) gene was used as a positive control for *R. solani* strains. **C** Pathogenicity assay of strains XY74, 190, and 190NV. Mycelium plugs of strains 190, XY74, and 190NV were inoculated on the detached rice leaves at 28 ​°C and continuously observed until 72 ​h post-inoculation (hpi). Pictures were taken each 24 ​h.

*R. solani* XY74 endorses a growth rate of 1.95 ​cm/day, while strain 190, a strain lacking RsAEV1 and RsFV4, endorses 2.25 ​cm/day. To investigate the effects of RsAEV1 and RsFV4 on its host *R. solani* strain XY74, hyphal tip isolation and protoplast regeneration were conducted to eliminate RsAEV1 and RsFV4. However, attempts to cure XY74 via both hyphal tipping culture and protoplast regeneration were unsuccessful. To trigger the steady-state level of transmissibility of abnormal traits in RsAEV1 and RsFV4, we utilized dual-culture approaches using the strains XY74 (donor) and 190 (recipient) on a PDA medium (Fig. 4). RsAEV1 and RsFV4 transmissions were confirmed using an RT-PCR approach. The newly obtained isolates (190NV) from strain 190, which received both RsAEV1 and RsFV4, displayed a phenotypic colony and *in-vivo* growth level indistinguishable from the parental strain 190 (Fig. 4A). These results showed that RsAEV1 and RsFV4 did not exert a discernible impact on the growth and colony morphology of their hosts. Furthermore, a virulence test on detached rice leaves was conducted to determine whether RsAEV1 and RsFV4 were associated with hypovirulence. The isolate 190NV continued to exhibit discernible lesions on the detached rice leaves maintained at 28 ​°C for 72 ​h post-inoculations (Fig. 4C), suggesting that RsAEV1 and RsFV4 did not exert apparent effects on host virulence.

## 4 Discussion

A previous study has described novel fusariviruses that infect the *R. solani* anastomosis group AG2-2LP (Picarelli et al., 2019). Sequence analysis and phylogenetic assessment have helped determine that RsFV4 should be classified as a member of the proposed Fusariviridae family. To the best of our knowledge, no fusarivirus has been previously reported to infect *R. solani* AG-1IA. Here described a monopartite (+)ssRNA fusarivirus, RsFV4, that infected the plant pathogenic fungus *R. solani* AG-1IA. Importantly, RsFV4 is the first well-characterized fusarivirus that infects *R. solani*, with helicases related to those present in fusariviruses, hypoviruses, and potyviruses. The (+)ssRNA fusarivirus RsFV4 was clustered with these viruses and presented an evolutionary link between mycoviruses and plant viruses or between mycoviruses, hence implying the existence of ancient relationships. Virus transmission among plants and fungi is a feature of the evolutionary history of several virus families (Roossinck, 2019). Plausible evidence of cross-kingdom virus transmission relies on results obtained via phylogenetic and field studies. Nevertheless, experimental evidence suggests its occurrence, which is limited to anastomoses. For example, Sclerotinia sclerotiorum mycoreovirus 4 can suppress host non-self recognition and allow the horizontal spread of heterologous viruses (Wu et al., 2017).

RNA viruses code for a single RdRp that plays an important role in viral genome transcription and replication (Shu and Gong, 2016). Phylogenetic analysis results helped divide the (+)ssRNA RdRp domain into three supergroups (I, II, and III), wherein supergroup I contained the potyviruses (Koonin, 1991). RsFV4 encodes an RdRp that is related to fusariviruses. An RdRp topology tree clustered RsFV4 in a well-supported clade (Group V) within fusariviruses and suggested its distant relation to hypoviruses and potyviruses (Fig. 3A). This is consistent with the hypothesis that fusariviruses are phylogenetically related to *Hypoviridae* and *Potyviridae* families, based on RdRp-based dendrograms (Kwon et al., 2007; Zhang et al., 2014). RdRp encompasses universal sequence motifs (I–VIII) found in all (+)ssRNA viruses (Koonin et al., 1993). However, only three motifs (IV, V, and VI) demonstrated complete and unequivocal conservation across the entire class, with six invariant amino acid residues (Koonin et al., 1993). Consistently, ORF2-encoded RsFV4 conserved motifs IV, V, and VI accommodate the invariant residues that were found in (+)ssRNA RdRp (Supplementary Fig. S1).

Helicases are ubiquitous proteins, and include necessary conserved enzymes in eukaryotes and prokaryotes (Cordin et al., 2006). The helicase is divided into six superfamilies (SFI to SFVI) based on their specific domain motifs. The majority of known RNA helicases belong to SFII, which comprises several viral families (Tanner and Linder, 2001; Cordin et al., 2006). Although viruses that contain two helicases have been previously reported, the presence of two helicases is uncommon in mycoviruses. Some endornaviruses and the recently reported hypovirus (Rhizoctonia solani hypovirus 2) also have two helicases belonging to different SFs (Abdoulaye et al., 2021; Tuomivirta et al., 2009). That two helicases in a single virus belong to the same superfamily were also reported, for instance, tymo-like viruses that encodes two putative helicases of SFI (Koonin et al., 1993), where the first helicase is involved in genome replication, while the second (or accessory) helicase demonstrates the evolution of additional biological functions, and is implicated in viral silencing and/or inter-cell movement in plants (Koonin et al., 1993; Morozov and Solovyev, 2015). Notably, recent studies have reported fusariviruses genomes containing two partial helicase domains (Picarelli et al., 2019). In the present study, we found that both Hel1 and Hel2 of RsFV4 belong to SFII and are located in different ORFs (ORF1 and ORF3, respectively), which were significantly different from all known mycoviruses that contain two helicases. The phylogenetic analysis of helicases and their relatives clustered Hel1 and Hel2 in two distantly related clades (Fig. 3B). Unexpectedly, Hel1 formed an independent clade with RsFV1, RsFV2, and RsHV3, related to potyviruses and hypoviruses. Nonetheless, Hel2 was grouped with other reported fusariviruses but was clustered separately with RsFV1, RsFV2, and RsFV3 (Fig. 3). Those results indicate that Hel1 of RsFV4 and its relatives is might have acquired from other mycoviruses (for example, hypoviruses), or a plant virus (such as potyviruses); additionally, this also suggests that Hel1 may be an accessory helicase.

Previous studies have shown that hypoviruses share common ancestry with plant potyviruses (Koonin et al., 1991), and fusariviruses share common ancestry with hypoviruses (Zhang et al., 2014). The presence of ORF1-encoded Hel1 in plant viruses suggests that RsFV4 with its fungi host may have previously infected plants. Therefore, its presence may indicate the occurrence of interaction between fusariviruses (mycoviruses) and potyviruses (plant viruses) or hypoviruses (mycoviruses). The significant interactions between plant and mycoviruses may be attributed partly to the diverse and evolutionarily ancient symbiotic relationships of terrestrial plants with fungi, which range from promotion of nutrient absorption to the development of stress tolerance (Malloch et al., 1980; Redman et al., 2002; Rodriguez and Redman, 2008; Bonfante and Genre, 2010).

RsFV4 contains four ORFs, together with RsFV1, and RsFV2. Notably, fusarivirurses exhibited variation in genome organization (Fig. 1E). Firstly, FgFV1 contains three putative ORFs of unknown function, while RsFV4, RsFV1, and RsFV2 contain two ORFs of unknown function. RdRp and helicase are common to all fusariviruses, and may be necessary for their replication. The presence of helicases in *R. solani* fusariviruses suggests that they are crucial for their replication. In contrast, the accessory helicases, so far, are present only in *R. solani* fusariviruses, and thus may not be necessary for mycoviruses replication and have evolved unknown biological functions for fusariviruses in *R. solani*. Members within *Hypoviridae* family generally contain a single large ORF (Smart et al., 1999; Linder-Basso et al., 2005). The genome organizations of fusariviruses and hypoviruses are different, but exhibit the common presence of the helicase and RdRp domains. Helicase and RdRp identities of *Hypoviridae* and RsFV4 showed moderate sequence identity. Moreover, *Potyviridae* contains the largest plant RNA virus family and is generally mono-segmented except *Bymovirus* (Wylie et al., 2017). *Potyviridae* contains one or two ORF(s) that encodes a polyprotein including RdRp, Hel, CP, P3, Vpg, and Pro (Wylie et al., 2017). Hypoviruses and fusariviruses are monopartite, whereas *Potyviridae* members are mono or bipartite (Wylie et al., 2017). Nevertheless, these families have RdRp and Hel in common, where potyvirus Hel accommodates larger amino acids.

## 5 Conclusions

In summary, two novel mycoviruses (RsFV4 and RsAEV1) co-infecting the plant pathogenic fungus *R. solani* were identified. Accessory helicases in *R. solani* fusariviruses evolutionarily link fusariviruses, hypoviruses, and potyviruses. It would be interesting to determine the function of accessory helicases in mycoviruses and the direction of helicase transmission between mycoviruses and plants or mycoviruses.

## Data availability

The complete sequences of RsAEV1 and RsFV4 genome have been deposited in GenBank (https://www.ncbi.nlm.nih.gov/genbank/) under the accession number MW149058 and MW149059, respectively.

## Ethics statement

This article does not contain any studies with human or animal subjects performed by any of the authors.

## Author contribution

Assane Hamidou Abdoulaye: conceptualization, writing – original draft, writing - review&editing, data curation, methodology. Jichun Jia: methodology, data curation. Aqleem Abbas: methodology Du Hai: methodology, data curation. Jiasen Cheng: investigation, methodology. Yanping Fu: investigation, methodology. Yang Lin: investigation, methodology. Daohong Jiang: supervision, conceptualization, investigation, writing - review&editing. Jiatao Xie: supervision, conceptualization, investigation, writing - review&editing, methodology, data curation. All authors listed have made a direct, substantial and intellectual contribution to this work, therefore, approved it for publication.

## Conflict of interest

The authors declare that they have no conflict of interest.

## Appendix A Supplementary data

figs1 figs1

## References

[bib1] Abdoulaye A.H., Hai D., Tang Q., Jiang D., Fu Y., Cheng J., Lin Y., Li B., Kotta-Loizou I., Xie J. (2021). Two distant helicases in one mycovirus: evidence of horizontal gene transfer between mycoviruses, coronaviruses and other nidoviruses. Virus Evol..

[bib2] Ballut L., Marchadier B., Baguet A., Tomasetto C., Séraphin B., Le Hir H. (2005). The exon junction core complex is locked onto RNA by inhibition of eIF4AIII ATPase activity. Nat. Struct. Mol. Biol..

[bib3] Bonfante P., Genre A. (2010). Mechanisms underlying beneficial plant-fungus interactions in mycorrhizal symbiosis. Nat. Commun..

[bib4] Bowers H.A., Maroney P.A., Fairman M.E., Kastner B., Lührmann R., Nilsen T.W., Jankowsky E. (2006). Discriminatory RNP remodeling by the DEAD-box protein DED1. RNA.

[bib5] Bräutigam A., Mullick T., Schliesky S., Weber A.P. (2011). Critical assessment of assembly strategies for non-model species mRNA-Seq data and application of next-generation sequencing to the comparison of C3 and C4 species. J. Exp. Bot..

[bib6] Capella-Gutiérrez S., Silla-Martínez J.M., Gabaldón T. (2009). trimAl: a tool for automated alignment trimming in large-scale phylogenetic analyses. Bioinformatics.

[bib7] Castanho B., Butler E., Shepherd R. (1978). The Association of double-stranded-RNA with *Rhizoctonia* decline. Phytopathology.

[bib8] Chu W.K., Hickson I.D. (2009). RecQ helicases: multifunctional genome caretakers. Nat. Rev. Cancer.

[bib9] Cordin O., Banroques J., Tanner N.K., Linder P. (2006). The DEAD-box protein family of RNA helicases. Gene.

[bib10] Cui S., Eisenächer K., Kirchhofer A., Brzózka K., Lammens A., Lammens K., Fujita T., Conzelmann K.K., Krug A., Hopfner K.P. (2008). The C-terminal regulatory domain is the RNA 5′-triphosphate sensor of RIG-I. Mol. Cell.

[bib11] Donaire L., Pagán I., Ayllón M.A.J.V. (2016). Characterization of Botrytis cinerea negative-stranded RNA virus 1, a new mycovirus related to plant viruses, and a reconstruction of host pattern evolution in negative-sense ssRNA viruses. Virology.

[bib12] Fairman-Williams M.E., Guenther U.P., Jankowsky E. (2010). SF1 and SF2 helicases: family matters. Curr. Opin. Struct. Biol..

[bib13] Feng H., Sun Z., Li H., Qin P., Tang C., Fu R., Liu Y., Li P., Zheng A. (2012). Preparation, purification and regeneration optimizing research of protoplasts from *Rhizoctonia solani*. Afr. J. Microbiol. Res..

[bib14] Garg R., Patel R.K., Tyagi A.K., Jain M. (2011). De novo assembly of chickpea transcriptome using short reads for gene discovery and marker identification. DNA Res..

[bib15] Ghabrial S.A., Castón J.R., Jiang D., Nibert M.L., Suzuki N. (2015). 50-plus years of fungal viruses. Virology.

[bib16] Gorbalenya A.E., Koonin E.V. (1993). Helicases: amino acid sequence comparisons and structure-function relationships. Curr. Opin. Struct. Biol..

[bib17] Granato L.M., Picchi S.C., de Oliveira Andrade M., Takita M.A., de Souza A.A., Wang N., Machado M.A. (2016). The ATP-dependent RNA helicase HrpB plays an important role in motility and biofilm formation in Xanthomonas citri subsp. citri. BMC Microbiol..

[bib18] Halls C., Mohr S., Del C.M., Yang Q., Jankowsky E., Lambowitz A.M. (2007). Involvement of DEAD-box proteins in group I and group II intron splicing. Biochemical characterization of Mss116p, ATP hydrolysis-dependent and-independent mechanisms, and general RNA chaperone activity. J. Mol. Biol..

[bib19] He Y., Andersen G.R., Nielsen K.H. (2010). Structural basis for the function of DEAH helicases. EMBO Rep..

[bib20] Hrabáková L., Grum-Grzhimaylo A.A., Koloniuk I., Debets A.J., Sarkisova T., Petrzik K. (2017). The alkalophilic fungus *Sodiomyces alkalinus* hosts beta-and gammapartitiviruses together with a new fusarivirus. PLoS One.

[bib21] Jankowsky E., Gross C.H., Shuman S., Pyle A.M. (2001). Active disruption of an RNA-protein interaction by a DExH/D RNA helicase. Science.

[bib22] Jankowsky E. (2011). RNA helicases at work: binding and rearranging. Trends Biochem. Sci..

[bib23] Jiang D., Ghabrial S.A. (2004). Molecular characterization of Penicillium chrysogenum virus: reconsideration of the taxonomy of the genus *Chrysovirus*. J. Gen. Virol..

[bib24] Kalyaanamoorthy S., Minh B.Q., Wong T.K., Von Haeseler A., Jermiin L.S. (2017). ModelFinder: fast model selection for accurate phylogenetic estimates. Nat. Methods.

[bib25] Katoh K., Standley D.M. (2013). MAFFT multiple sequence alignment software version 7: improvements in performance and usability. Mol. Biol. Evol..

[bib26] Kearse M., Moir R., Wilson A., Stones-Havas S., Cheung M., Sturrock S., Buxton S., Cooper A., Markowitz S., Duran C., Thierer T. (2012). Geneious Basic: an integrated and extendable desktop software platform for the organization and analysis of sequence data. Bioinformatics.

[bib27] Koonin E.V., Dolja V.V., Morris T.J. (1993). Evolution and taxonomy of positive-strand RNA viruses: implications of comparative analysis of amino acid sequences. Crit. Rev. Biochem. Mol..

[bib28] Koonin E.V., Choi G.H., Nuss D.L., Shapira R., Carrington J.C. (1991). Evidence for common ancestry of a chestnut blight hypovirulence-associated double-stranded RNA and a group of positive-strand RNA plant viruses. Proc. Natl. Acad. Sci. U.S.A..

[bib29] Koonin E.V. (1991). The phylogeny of RNA-dependent RNA polymerases of positive-strand RNA viruses. J. Gen. Virol..

[bib30] Kwon S.J., Lim W.S., Park S.H., Park M.R., Kim K.H. (2007). Molecular characterization of a dsRNA mycovirus, Fusarium graminearum virus-DK21, which is phylogenetically related to hypoviruses but has a genome organization and gene expression strategy resembling those of plant potex-like viruses. Mol. Cell.

[bib31] Larsen N.B., Hickson I.D. (2013). RecQ helicases: conserved guardians of genomic integrity. Adv. Exp. Med. Biol..

[bib32] Linder-Basso D., Dynek J.N., Hillman B.I. (2005). Genome analysis of Cryphonectria hypovirus 4, the most common hypovirus species in North America. Virology.

[bib33] Liu C., Zeng M., Zhang M., Shu C., Zhou E. (2018). Complete nucleotide sequence of a partitivirus from Rhizoctonia solani AG-1 IA strain C24.. Viruses.

[bib34] Liu H., Fu Y., Jiang D., Li G., Xie J., Cheng J. (2010). Widespread horizontal gene transfer from double-stranded RNA viruses to eukaryotic nuclear genomes. J. Virol..

[bib35] Liu R., Cheng J., Fu Y., Jiang D., Xie J. (2015). Molecular Characterization of a novel positive-sense, single-stranded RNA mycovirus infecting the plant pathogenic fungus *Sclerotinia sclerotiorum*. Viruses.

[bib36] Liu W., Hai D., Mu F., Yu X., Zhao Y., He B., Xie J., Jiang D., Liu H. (2020). Molecular characterization of a novel fusarivirus infecting the plant-pathogenic fungus *Botryosphaeria dothidea*. Arch. Virol..

[bib37] Lyu R., Zhang Y., Tang Q., Li Y., Cheng J., Fu Y., Chen T., Jiang D., Xie J. (2018). Two alphapartitiviruses co-infecting a single isolate of the plant pathogenic fungus *Rhizoctonia solani*. Arch. Virol..

[bib38] Malloch D.W., Pirozynski K.A., Raven P.H. (1980). Ecological and evolutionary significance of mycorrhizal symbioses in vascular plants (a review). Proc. Natl. Acad. Sci. U.S.A..

[bib39] Minh B.Q., Schmidt H.A., Chernomor O., Schrempf D., Woodhams M.D., Von Haeseler A., Lanfear R. (2020). IQ-TREE 2: new models and efficient methods for phylogenetic inference in the genomic era. Mol. Biol. Evol..

[bib40] Morozov S.Y., Solovyev A.G. (2015). Phylogenetic relationship of some “accessory” helicases of plant positive-stranded RNA viruses: toward understanding the evolution of triple gene block. Front. Microbiol..

[bib41] Picarelli M.A.S., Forgia M., Rivas E.B., Nerva L., Chiapello M., Turina M., Colariccio A. (2019). Extreme diversity of mycoviruses present in isolates of *Rhizoctonia solani* AG2-2 LP from Zoysia japonica from Brazil. Front. Cell. Infect. Microbiol..

[bib42] Potgieter A., Page N., Liebenberg J., Wright I., Landt O., Van Dijk A. (2009). Improved strategies for sequence-independent amplification and sequencing of viral double-stranded RNA genomes. J. Gen. Virol..

[bib43] Redman R.S., Sheehan K.B., Stout R.G., Rodriguez R.J., Henson J.M. (2002). Thermotolerance generated by plant/fungal symbiosis. Science.

[bib44] Rodriguez R., Redman R.J. (2008). More than 400 million years of evolution and some plants still can't make it on their own: plant stress tolerance via fungal symbiosis. J. Exp. Bot..

[bib45] Rodriguez R.J., White J.F., Arnold A.E., Redman R.S. (2009). Fungal endophytes: diversity and functional roles. New Phytol..

[bib46] Roossinck M.J. (2019). Evolutionary and ecological links between plant and fungal viruses. New Phytol..

[bib47] Sankar S., Porter A.J. (1992). Point mutations which drastically affect the polymerization activity of encephalomyocarditis virus RNA-dependent RNA polymerase correspond to the active site of *Escherichia coli* DNA polymerase I. J. Biol. Chem..

[bib48] Shu B., Gong P. (2016). Structural basis of viral RNA-dependent RNA polymerase catalysis and translocation. Proc. Natl. Acad. Sci. U.S.A..

[bib49] Singleton M.R., Dillingham M.S., Wigley D.B. (2007). Structure and mechanism of helicases and nucleic acid translocases. Annu. Rev. Biochem..

[bib50] Smart C., Yuan W., Foglia R., Nuss D., Fulbright D., Hillman B. (1999). Cryphonectria hypovirus 3, a virus species in the family *Hypoviridae* with a single open reading frame. Virology.

[bib51] Son M., Yu J., Kim K.-H. (2015). Five questions about mycoviruses. PLoS Pathog..

[bib52] Su C., Chao Y.T., Alex Chang Y.C., Chen W.C., Chen C.Y., Lee A.Y., Hwa K.T., Shih M.C. (2011). De novo assembly of expressed transcripts and global analysis of the Phalaenopsis aphrodite transcriptome. Plant Cell Physiol..

[bib53] Tanner N.K., Linder P. (2001). DExD/H box RNA helicases: from generic motors to specific dissociation functions. Mol. Cell.

[bib54] Theuser M., Höbartner C., Wahl M.C., Santos K.F. (2016). Substrate-assisted mechanism of RNP disruption by the spliceosomal Brr2 RNA helicase. Proc. Natl. Acad. Sci. U.S.A..

[bib55] Tuomivirta T.T., Kaitera J., Hantula J. (2009). A novel putative virus of Gremmeniella abietina type B (Ascomycota: Helotiaceae) has a composite genome with endornavirus affinities. J. Gen. Virol..

[bib56] Wang W. (2015). International Conference on Computer and Computing Technologies in Agriculture.

[bib57] Wu S., Cheng J., Fu Y., Chen T., Jiang D., Ghabrial S.A., Xie J. (2017). Virus-mediated suppression of host non-self recognition facilitates horizontal transmission of heterologous viruses. PLoS Pathog..

[bib58] Wylie S.J., Adams M., Chalam C., Kreuze J., López-Moya J.J., Ohshima K., Praveen S., Rabenstein F., Stenger D., Wang A., Zerbini F.M. (2017). ICTV virus taxonomy profile: *Potyviridae*. J. Gen. Virol..

[bib59] Xia Y., Fei B., He J., Zhou M., Zhang D., Pan L., Li S., Liang Y., Wang L., Zhu J., Li P. (2017). Transcriptome analysis reveals the host selection fitness mechanisms of the *Rhizoctonia solani* AG-1IA pathogen. Sci. Rep..

[bib60] Xie J., Wei D., Jiang D., Fu Y., Li G., Ghabrial S., Peng Y. (2006). Characterization of debilitation-associated mycovirus infecting the plant-pathogenic fungus *Sclerotinia sclerotiorum*. J. Gen. Virol..

[bib61] Yang Q., Jankowsky E. (2005). ATP-and ADP-dependent modulation of RNA unwinding and strand annealing activities by the DEAD-box protein DED1. Biochemestry.

[bib62] Zhang M., Zheng L., Liu C., Shu C., Zhou E. (2018). Characterization of a novel dsRNA mycovirus isolated from strain A105 of *Rhizoctonia solani* AG-1 IA. Arch. Virol..

[bib63] Zhang R., Liu S., Chiba S., Kondo H., Kanematsu S., Suzuki N. (2014). A novel single-stranded RNA virus isolated from a phytopathogenic filamentous fungus, *Rosellinia necatrix*, with similarity to hypo-like viruses. Front. Microbiol..

[bib64] Zhang S., Grosse F. (2004). Multiple functions of nuclear DNA helicase II (RNA helicase A) in nucleic acid metabolism. Acta Biochim. Biophys. Sin..

[bib65] Zheng L., Liu H., Zhang M., Cao X., Zhou E. (2013). The complete genomic sequence of a novel mycovirus from *Rhizoctonia solani* AG-1 IA strain B275. Arch. Virol..

[bib66] Zheng L., Zhang M., Chen Q., Zhu M., Zhou E. (2014). A novel mycovirus closely related to viruses in the genus Alphapartitivirus confers hypovirulence in the phytopathogenic fungus *Rhizoctonia solani*. Virology.

[bib68] Zheng L., Shu C., Zhang M., Yang M., Zhou E. (2019). Molecular characterization of a novel endornavirus conferring hypovirulence in rice sheath blight fungus *Rhizoctonia solani* AG-1 IA strain GD-2. Viruses.

[bib67] Zhong J., Chen C.Y., Gao B.D. (2015). Genome sequence of a novel mycovirus of *Rhizoctonia solani*, a plant pathogenic fungus. Virus Gene..

