# Supplementary figures and images for: Fusarivirus accessory helicases present an evolutionary link for viruses infecting plants and fungi
Source: Virol Sin. 2022 Mar 18;37(3):427–36. doi: 10.1016/j.virs.2022.03.010 (PMC9243621; doi:10.1016/j.virs.2022.03.010)

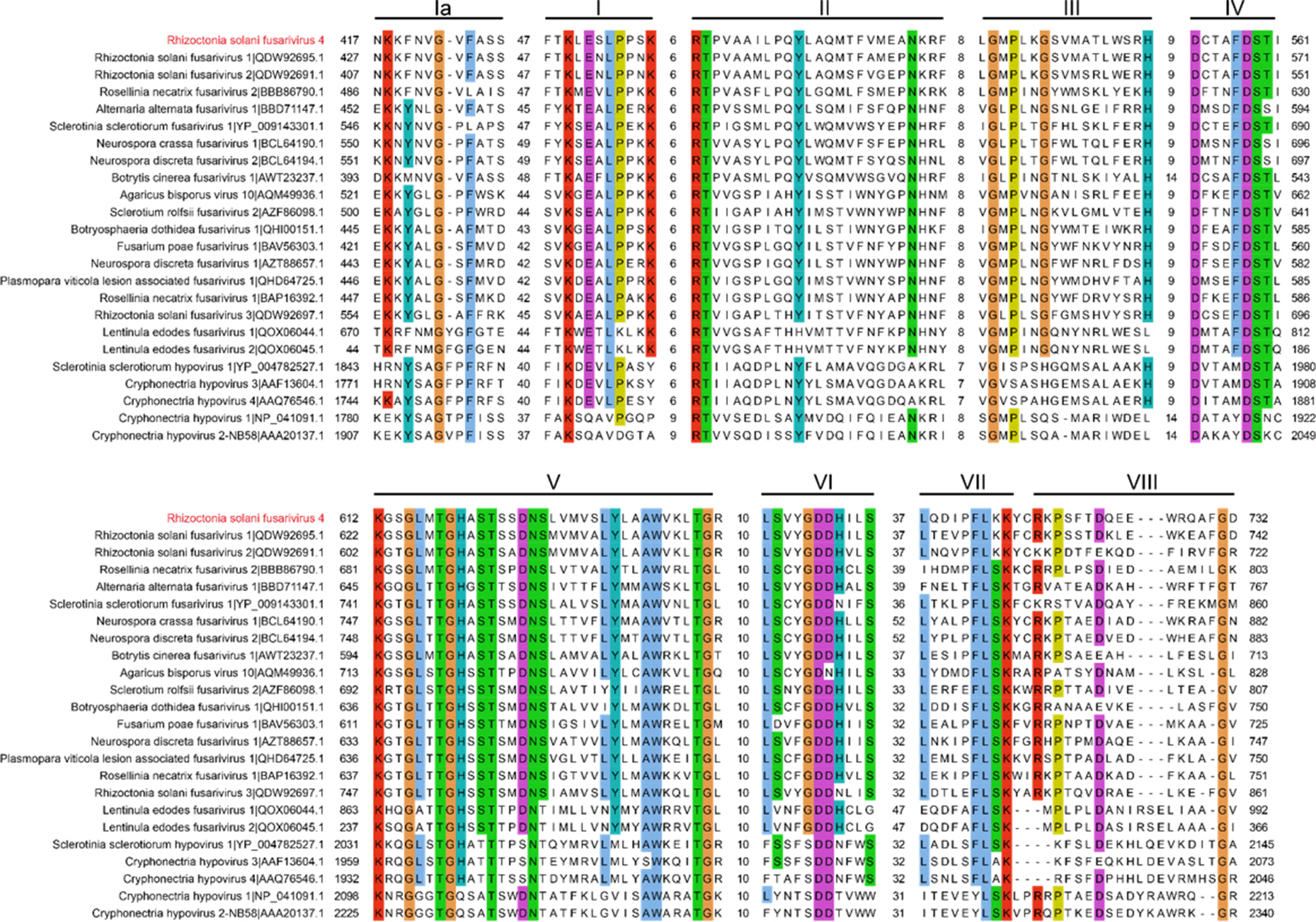

Supplement: figs1 [file figs1.jpg]

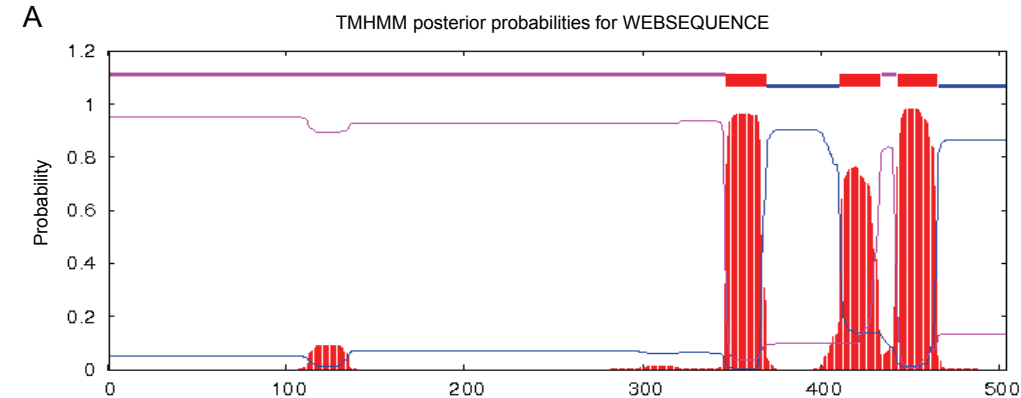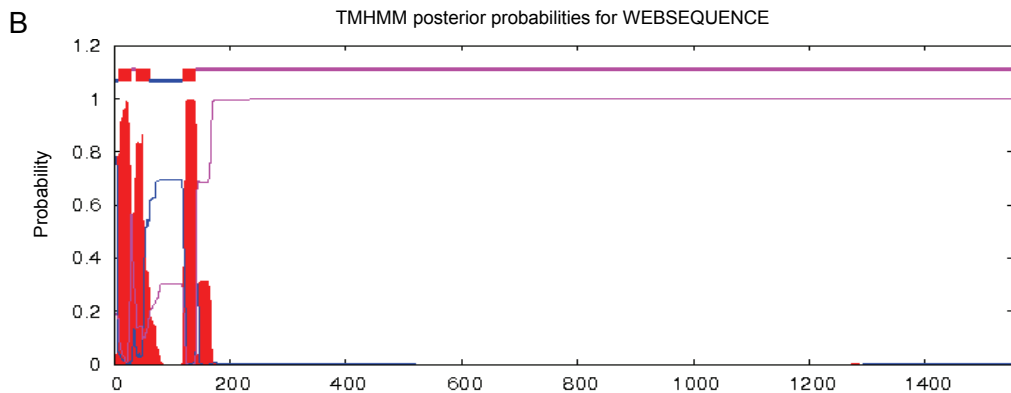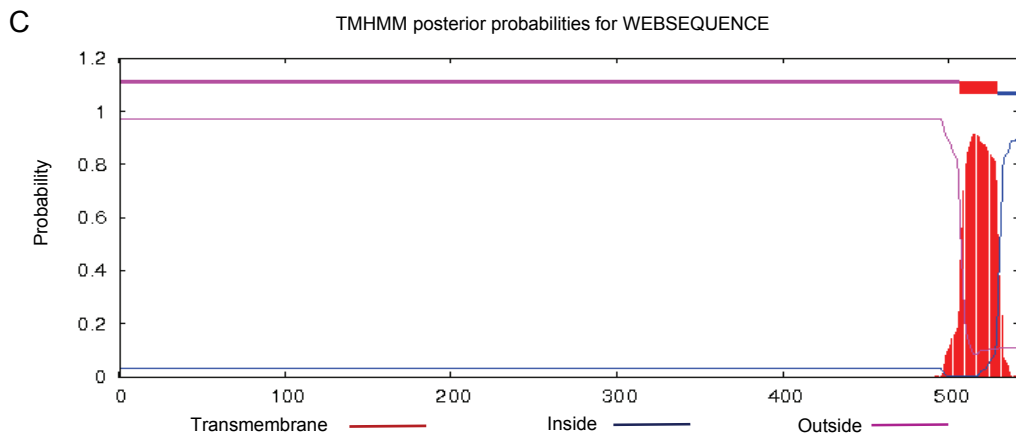

Supplement: Multimedia component 1 [file mmc1.pdf]
