# Supplementary material for: Fusarivirus accessory helicases present an evolutionary link for viruses infecting plants and fungi
Source: Virol Sin. 2022 Mar 18;37(3):427–36. doi: 10.1016/j.virs.2022.03.010 (PMC9243621; doi:10.1016/j.virs.2022.03.010)
Supplement: Multimedia component 2 [file mmc2.docx]

**Virologica Sinica**

**Supplementary Data**

**Fusarivirus accessory helicases present an evolutionary link for viruses infecting plants and fungi**

Assane Hamidou Abdoulaye ^a, b, c^, Jichun Jia ^a, b, c^, Aqleem Abbas ^a, b, c^, Du Hai ^a, b, c^, Jiasen Cheng ^a, b^, Yanping Fu ^b^, Yang Lin ^b^, Daohong Jiang ^a, b, c^, Jiatao Xie ^a, b, c, *^

^a^ State Key Laboratory of Agricultural Microbiology, Huazhong Agricultural University, Wuhan 430070, China;

^b^ Hubei Key Laboratory of Plant Pathology, College of Plant Science and Technology, Huazhong Agricultural University, Wuhan 430070, China;

^c^ Hubei Hongshan Laboratory, Wuhan 430070, China

^*^ Corresponding author

Email address: jiataoxie@mail.hzau.edu.cn (J. Xie)

ORCID : 0000-0003-1961-0338

**Supplementary materials**

**
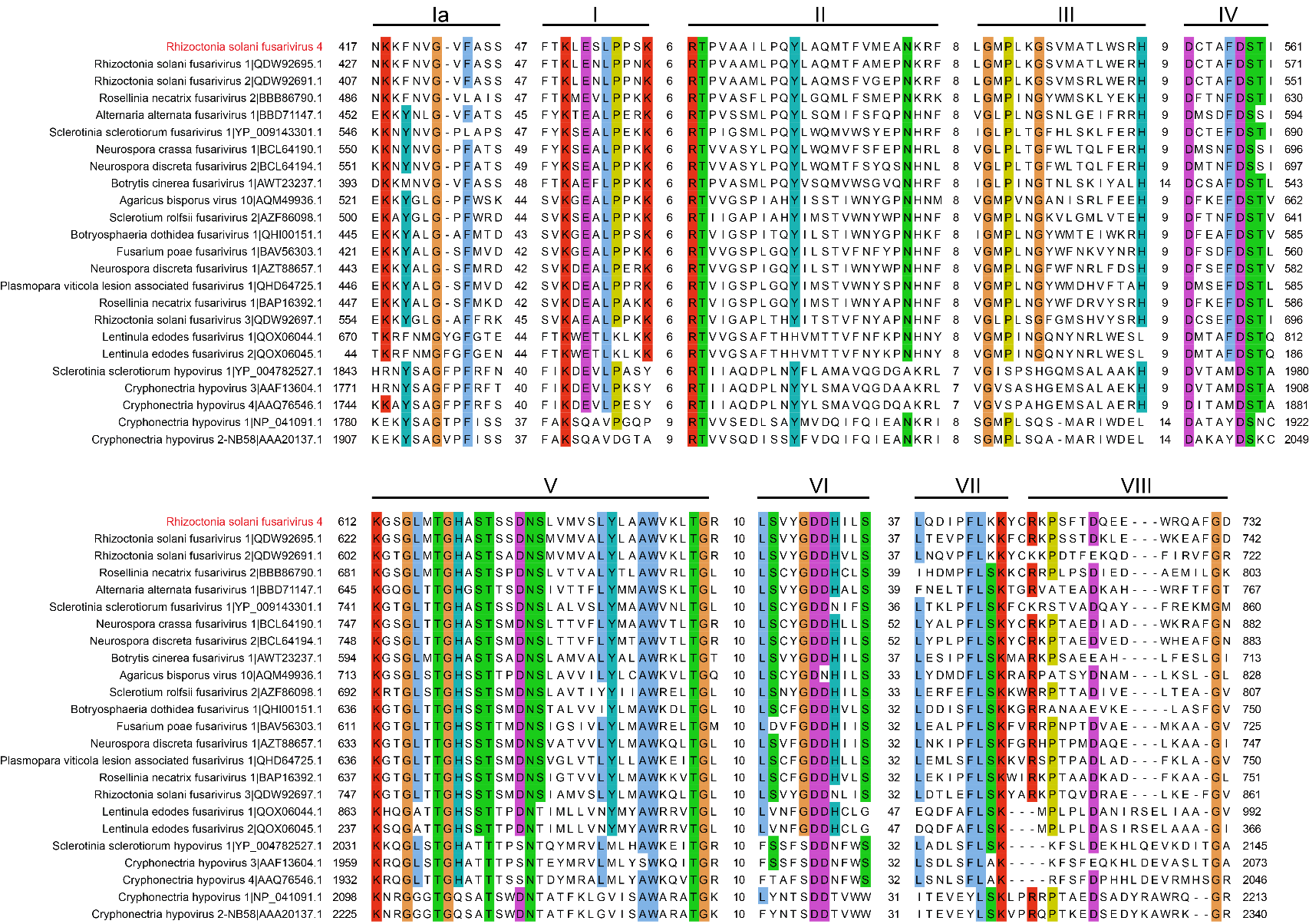
**

**Fig. S1:** Amino acid sequences alignment of the conserved domains of RdRp in RsFV4 and its related members within the proposed family “*Fusariviridae*” and *Hypoviridae*. The default color scheme for percentage identity in the Jalview program was used. Numbers I–VIII refer to the eight conserved motifs characteristic of RNA viruses RdRp. The numbers within sequence correspond to the number of amino acid residues separating the motifs. The numbers of two flanks of sequences refer to the region of RdRp domain in polyprotein encoded by mycoviruses referenced.


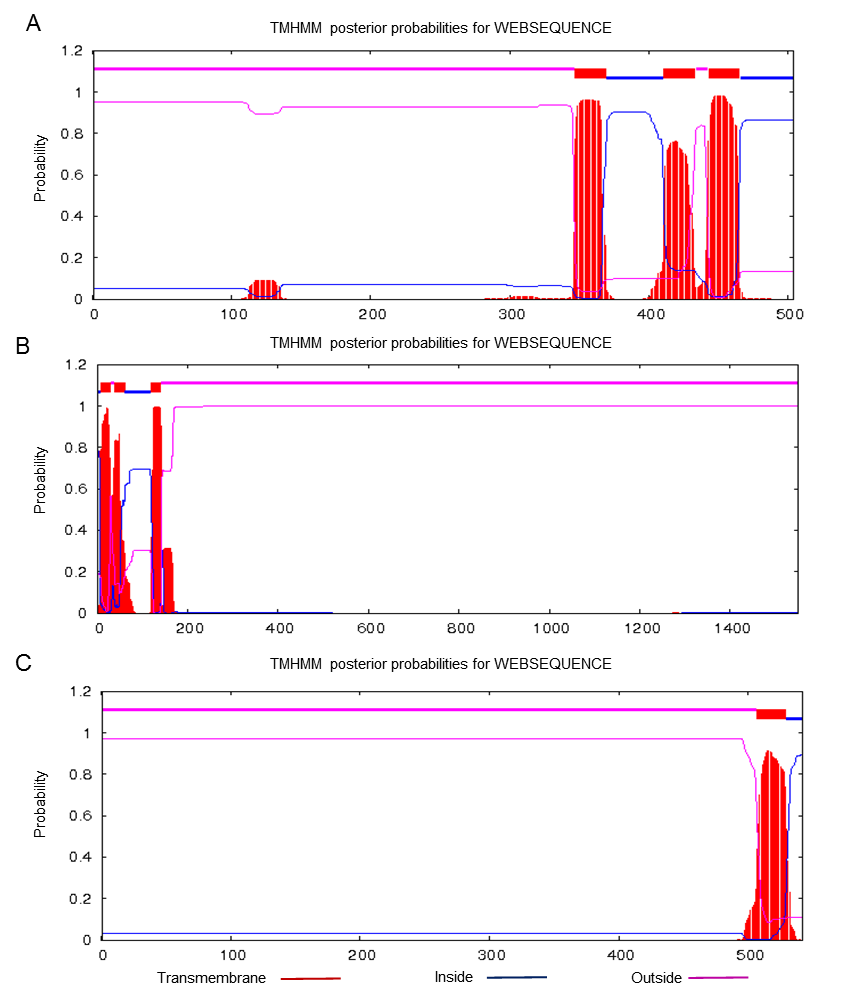


**Fig. S2:** Transmembrane regions in RsFV4 encoded proteins were predicted. **A** ORF2 encoded proteins contains three transmembrane regions at the C-terminal region. **B** Protein encoded by ORF3 has two transmembrane regions at the N-terminal region. **C** Protein encoded by ORF4 contains a single transmembrane region at the C-terminal region. Transmembrane region was predicted using the online program TMHMM-2.0 (https://services.healthtech.dtu.dk/service.php?TMHMM-2.0).

**Table S1.** Primer names and sequences were used to amplify RsFV4 and the amplicon size.

| **Primer name** | **Primer sequences (5**′ **– 3**′**)** | **Amplicon size (nts)** |
| --- | --- | --- |
| F1 | TCTTGTTTCCAGCGAGACCC | 1149 |
| R1 | GTTGGTGTGACGCTACCTGA |  |
| F2 | AGGCCACAAACGAAACGTCCGA | 1294 |
| R2 | TTCGCCGTTTGGCAGGGTGT |  |
| F3 | ACACCCTGCCAAACGGCGAAA | 1383 |
| R3 | TGTGGCCTTGAAGCTCGGTCA |  |
| F4 | TCCTCTGCAGATGTGGGGATGACA | 1021 |
| R4 | ACTGCCGCTGGTGCTTTAGGT |  |
| F5 | TGTGATGGACACTCTGGAGGA | 1912 |
| R5 | GGCTTCCTCCTCTTCATCGT |  |
| F6 | TTGTGACGAGCCCCTTCCTA | 1020 |
| R6 | GTCGCCCCAACAACTCAAAG |  |
| F7 | ACGATGAAGAGGAGGAAGCC | 1963 |
| R7 | AGGCCACAATGGCATCAGTA |  |
| F8 | CAGGTGTCGAGGCGCTTTTA | 1972 |
| R8 | TTCAAAATTGCCCATGCGGG |  |
| F9 | GATGGGCAAAGGGTTCCTCA | 1940 |
| R9 | TGCAAGTGACCCAAGGTTGT |  |
| F10 | TGGGAAAGTCATGGCTGATGT | 1982 |
| R10 | TTCCTGAGGGACGGCTTTTG |  |
| F11 | CTGCAGATGTGGGGATGACA | 1910 |
| R11 | TTCGCATTAGTGCCAGTGTT |  |
| F12 | CCACCACCTGATGTCCCTAC | 1154 |
| R12 | AAACTTCCGTTCCAGGACGC |  |

**Table S2.** The information of the viruses used for phylogenetically analysis in this study.

| **Virus name** | **Abb.** | **Accession number** |
| --- | --- | --- |
| Rhizoctonia solani fusarivirus 4 | RsFV4 | MW149059 |
| Rhizoctonia solani fusarivirus 3 | RsFV3 | MK558258 |
| Rhizoctonia solani fusarivirus 2 | RsFV2 | MK558256 |
| Rhizoctonia solani fusarivirus 1 | RsFV1 | QDW92695 |
| Rhizoctonia solani hypovirus 3 | RsHV3 | MK558255 |
| Cryphonectria hypovirus 1 | CHV1 | NC_001492 |
| Cryphonectria hypovirus 2 | CHV2 | NC_003534 |
| Cryphonectria hypovirus 3 | CHV3 | NC_000960 |
| Cryphonectria hypovirus 4 | CHV4 | NC_006431 |
| Rosellinia necatrix hypovirus 1 | RnHV1 | NC_036590 |
| Sclerotinia sclerotiorum hypovirus 2 | SsHV2 | NC_022896 |
| Sclerotinia sclerotiorum fusarivirus 1 | SsFV1 | NC_027208 |
| Rosellinia necatrix fusarivirus 1 | RnFV1 | NC_024485 |
| Sclerotinia homoeocarpa fusarivirus 1 | ShFV1 | MK279505 |
| Alternaria brassicicola fusarivirus 1 | AbFV1 | NC_029056 |
| Neurospora crassa fusarivirus 1 | NcFV1 | LC586025 |
| Nigrospora oryzae fusarivirus 1 | NoFV1 | NC_031960 |
| Sclerotium rolfsii fusarivirus 1 | SrFV1 | MH766491 |
| Sclerotium rolfsii fusarivirus 2 | SrFV2 | MH766492 |
| Auricularia heimuer fusarivirus 1 | AhFv1 | MT232758 |
| Fusarium poae fusarivirus 1 | FpFV1 | NC_030868 |
| Aspergillus ellipticus fusarivirus 1 | AeFV1 | MK279500 |
| Neofusicoccum luteum fusarivirus 1 | NlFV1 | KY906214 |
| Neurospora discreta fusarivirus 1 | NdFV1 | MK279503 |
| Botryosphaeria dothidea fusarivirus 1 | BdFV1 | QHI00151 |
| Agaricus bisporus virus 10 | AbV10 | AQM49937 |
| Agaricus bisporus virus 11 | AbV11 | AQM49938 |
| Monilinia fusarivirus G | MfFV-G | QED42908 |
| Plasmopara viticola lesion associated fusarivirus 2 | PvLaFV2 | QHD64732 |
| Gaeumannomyces tritici fusarivirus 1 | GtFV1 | AZT88652 |
| Plasmopara viticola lesion associated fusarivirus 1 | PvLaFV2 | QHD64725 |
| Plasmopara viticola lesion associated fusarivirus 3 | PvLaFV3 | QHD64735 |
| Sodiomyces alkalinus fusarivirus 1 | SaFV1 | ATP75827 |
| Rutstroemia firma fusarivirus 1 | RfFV1 | AZT88659 |
| Bipolaris oryzae hypovirus 1 | BoHV1 | MH316122 |
| Macrophomina phaseolina hypovirus 1 | MpHV1 | NC_003534 |
| Fusarium graminearum hypovirus 1 | FgHV1 | NC_023680 |
| Alternaria alternata hypovirus 1 | AaHV1 | MK189193 |
| Fusarium sambucinum hypovirus 1 | FsHV1 | LC596823 |
| Botrytis cinerea hypovirus 1 | BcHV1 | QBA69887 |
| Valsa ceratosperma hypovirus 1 | VcHV1 | NC_017099 |
| Phomopsis longicolla hypovirus | PlHV | NC_024685 |
| Sclerotinia sclerotiorum hypovirus 1 | SsHV1 | NC_015939 |
| Botrytis cinerea hypovirus 1 | BcHV1 | NC_037659 |
| Monilinia hypovirus A | MfHVA | MK231061 |
| Rhizoctonia solani hypovirus 1 | RsHV1 | MK558259 |
| Rosellinia necatrix hypovirus 2 | RnHV2 | LC333749 |
| Agaricus bisporus virus 2 | AbHV2 | KY357507 |
| Sclerotium rolfsii hypovirus 1 | SrHV1 | MH037014 |
| Sclerotinia homoeocarpa hypovirus 1 | ShHV1 | MK279473 |
| Fusarium poae hypovirus 1 | FpHV1 | LC150612 |
| Fusarium langsethiae hypovirus 1 | FlHV1 | NC_032212 |
| Fusarium graminearum hypovirus 2 | FgHV2 | NC_026813 |
| Agaricus bisporus virus 2 | AbV2 | KY357507 |
| Entoleuca hypovirus 1 | EnHV1 | MF536691 |
| Triticum mosaic virus | TriMV | NC_012799 |
| Tomato mild mottle virus | TMMoV | NC_038920 |
| Oat necrotic mottle virus | ONMV | NC_005136 |
| Bellflower veinal mottle virus | BVMoV | NC_039002 |
| Areca palm necrotic spindle-spot virus | ANSSV | NC_040836 |
| Cardamom mosaic virus | CdMV | NC_039088 |
| Barley yellow mosaic virus | BaYMV | NC_002990 |
| Oat mosaic virus | OMV | NC_004016 |
| Tobacco vein banding mosaic virus | TVBMV | NC_009994 |
| Hordeum mosaic virus | HoMV | NC_005904 |
| Beet mosaic virus | BtMV | NC_005304 |
| Bean yellow mosaic virus | BYMV | NC_003492 |
| Potato virus A | PVA | NC_004039 |
| Hyacinth mosaic virus | HyaMV | NC_037051 |
| Tobacco vein mottling virus | TVMV | NC_001768 |
| Papaya ringspot virus | PRSV | NC_001785 |
| Cucurbit vein banding virus | CVBV | NC_035134 |
| Verbena virus Y | VVY | NC_010735 |
| Blackberry virus Y | BlVY | NC_008558 |
| Bean common mosaic virus | BCMV | NC_003397 |
| Saffron latent virus | SaLV | NC_036802 |
| Pepper yellow mosaic virus | PepYMV | NC_014327 |
| Tobacco mosqueado virus | TMosqV | NC_030118 |
| Panax virus Y | PanVY | NC_014252 |
| Sclerotinia sclerotiorum endornavirus 1 | SsEV1 | NC_021706 |
| Botrytis cinerea betaendornavirus 1 | BcEV1 | NC_031752 |
| Gremmeniella abietina betaendornavirus 1 | GaEV1 | NC_007920 |
| Sclerotinia minor betaendornavirus 1 | SmEV1 | NC_040631 |
| Alternaria brassicicola betaendornavirus 1 | AbEV1 | NC_026136 |
| Rosellinia necatrix betaendornavirus 1 | RnEV1 | NC_030938 |
| Rhizoctonia solani endornavirus 4 | RsEV4 | MK393902 |
| Lagenaria siceraria alphaendornavirus | LsEV | NC_023641 |
| Cucumis melo alphaendornavirus | CmEV | NC_029064 |
| Yerba mate alphaendornavirus | YmEV | NC_024455 |
| Phaseolus vulgaris alphaendornavirus 1 | PvEV1 | NC_039217 |
| Oryza sativa alphaendornavirus | OsEV | NC_007647 |
| Persea americana alphaendornavirus 1 | PaEV1 | NC_016648 |
| Helicobasidium mompa alphaendornavirus 1 | HmEV1 | NC_013447 |
| Grapevine endophyte alphaendornavirus | GEEV | NC_019493 |
| Erysiphe cichoracearum alphaendornavirus | EcEV | NC_029095 |
| Rhizoctonia cerealis alphaendornavirus 1 | RcEV1 | NC_022619 |
| Rhizoctonia solani alphaendornavirus 2 | RsEV2 | KT823701 |
| Vicia faba alphaendornavirus | VfEV | NC_007648 |
| Hot pepper alphaendornavirus | HPEV | NC_027920 |
| Bell pepper alphaendornavirus | BPEV | NC_039216 |
| Phaseolus vulgaris alphaendornavirus 2 | PvEV2 | NC_038422 |
| Winged bean alphaendornavirus 1 | WBEV1 | NC_031336 |
| Hordeum vulgare alphaendornavirus | HvEV | NC_031336 |
| Cluster bean alphaendornavirus 1 | CBEV1 | NC_040825 |
| Helianthus annuus alphaendornavirus | HaEV | NC_040799 |
| Agaricus bisporus endornavirus 1 | AbV1 | KY357509 |
| Phytophthora alphaendornavirus 1 | PEV1 | NC_007069 |
| Phaseolus vulgaris alphaendornavirus 3 | PvEV3 | NC_040558 |
